# Comparative Analyses of Exoproteinases Produced by Three Phytopathogenic Microorganisms

**DOI:** 10.4061/2011/947218

**Published:** 2011-12-14

**Authors:** Tatiana A. Valueva, Natalia N. Kudryavtseva, Alexis V. Sof'in, Tatiana A. Revina, Ekaterina L. Gvozdeva, Elena V. Ievleva

**Affiliations:** A.N. Bach Institute of Biochemistry, Russian Academy of Sciences, Leninsky Prospect 33-2, Moscow 119071, Russia

## Abstract

Proteinases secreted by the oomycete *Phytophthora infestans* (Mont.) de Bary, *Rhizoctonia solani,* and *Fusarium culmorum* belonging to different families of fungi have been studied to determine if the exoenzyme secretion depends on the environmental conditions and the phylogenetic position of the pathogen. The substrate specificity of the extracellular proteinases of *F. culmorum*, *R. solani*, and *P. infestans* and their sensitivity to the action of synthetic and protein inhibitors suggest that they contain trypsin-like and subtilisin-like enzymes regardless of culture medium composition. The relation of trypsin-like and subtilisin-like enzymes is dependent on the culture medium composition, especially on the form of nitrogen nutrition, particularly in the case of the exoenzymes secreted by *R. solani*. Phylogenetic analyses have shown that the exoproteinase set of ascomycetes and oomycetes has more similarities than basidiomycetes although they are more distant relatives. Our data suggests that the multiple proteinases secreted by pathogenic fungi could play different roles in pathogenesis, increasing the adaptability and host range, or could have different functions in survival in various ecological habitats outside the host.

## 1. Introduction

Fungi and oomycetes are responsible for many of the most devastating plant diseases that lead to very significant losses in the agricultural sector worldwide. Approximately 100,000 species of fungi and oomycetes have been described, but only a very small proportion of these are pathogenic [1]. However, phylogenetic studies have shown that disease-causing pathogens are not necessarily closely related to each other. In fact, they are spread throughout all taxonomic groups of fungi, often showing a close evolutionary relationship to nonpathogenic species [2]. It therefore seems likely that phytopathogenicity has evolved as a trait many times during fungal and oomycete evolution [2]. A significant effort has been made into the identification of pathogenicity determinants such as individual genes that are essential for a pathogen to invade a host plant successfully but that are dispensable for saprophytic growth [3]. 

Despite the different origin and different site on the phylogenetic tree of the true fungi and oomycetes [4], it has been shown that a range of secreted proteins known as effectors are important for establishing infection of the host plant [2]. These secreted proteins can suppress plant defenses and subvert cellular processes to suit the needs of invading pathogens. They include a number of secreted proteinases, transcriptional factors, and components of signal transduction pathways. In fungi, the proteinases can play either a general nutritive role or specific roles in cell metabolism or as pathogenicity or virulence factors. In fungi aspartate, cysteine, metallo-, serine, and threonine proteinases, as well as uncharacterized classes of proteinases, have been identified [5].

A total of 282,061 predicted proteins were grouped into 23,724 clusters, and only 16 clusters contained proteins that were found in all 34 species of fungi but which were absent from some species of oomycetes [6]. This number of fungal-specific clusters is surprisingly low considering the phylogenetic distance between the oomycetes and fungi. Pathogenicity factors have been defined as genes that are essential for successful completion of the pathogenic life cycle but dispensable for saprophytic growth [6].

Fungal life style and environment determine the array of enzymes that are expressed. Fungi have many trophic relationships with different groups of organisms. Proteinases that are secreted by fungi (especially pathogens) allow them to adapt to different living conditions. Fungi have expanded their repertoire of peptidases throughout evolution to take advantages of different protein sources. Proteinases are divided into six main classes—serine, cysteine, threonine, aspartic, glutamic, and metallopeptidases, according to the nature of functional groups in the active center of the enzyme molecule. Extracellular proteolytic enzymes of the fungi are represented to a large degree by serine proteinases. There are two major families of serine proteinases that are present in fungi: subtilisin (S8) and chymotrypsin (S1) families [5]. The chymotrypsin family includes trypsins and chymotrypsins and most of these enzymes in fungi are trypsins. According to preliminary observations, the production of trypsin-like enzymes is characteristic of plant pathogens whereas extracellular endoproteolytic activity of saprotrophs is provided mainly with subtilisin-like enzymes [7–9].

The most essential enzymes for pathogenesis can well be those that allow the fungus to penetrate the protective cutin layer of the plant epidermis and disrupt the pectin matrix of the plant cell wall in which cellulose fibrils are embedded. There seems to be a number of proteinase and peptidase domains that are more common in the genomes of phytopathogens [6, 10]. Proteolysis is an essential part of many physiological processes in all living organisms. Proteinases are also implicated in many pathological conditions in animals and plants and are considered as important virulence factors of many pathogens, including viruses, bacteria, fungi, and parasites. Proteinases from microorganisms display many unique characteristics in terms of catalytic mechanism, substrate specificity, activation mechanism, thermostability, and pH optimum [11]. Fungi could represent another source of proteinases with novel characteristics, as proteinases play a major role in the physiology, morphogenesis, and metabolism of fungi. In view of these functions, proteinases of several catalytic classes have been identified as being secreted from mycelia. They include serine proteinases, most of which have been classified as subtilisins [12]. 

The purpose of this study was a comparative analysis of proteinases secreted by *Phytophthora infestans* (Mont.) de Bary, *Rhizoctonia solani,* and *Fusarium culmorum* that are causal agents of several potato diseases. The pathogenic microorganisms belong to different families of fungi and pseudofungi. We thought that the data obtained from this study may help clarify the question whether their composition depends on the phylogenetic position of the pathogen.

## 2. Materials and Methods

### 2.1. Organisms and Cultivation Methods

The isolates of *Phytophthora infestans *(Mont.) de Bary*, Rhizoctonia solani Kuhn* (*AG-3)* 153, and *Fusarium culmorum* (W. G. Sm.) Sacc. were supplied by Potato, Vegetable, and Fruit Scientific and Practical Center of the National Academy of Sciences of Belorussia. The cultures were maintained on oatmeal agar and stored at room temperature (21°C). Culture media were tested for suitability to give good growth as well as for adequate enzyme production. The following media were tested: (I) per 100 mL: KH_2_PO_4_ (0.15 g); MgSo_4_·7H_2_O_2_ (0.025 g); FeSO_4_·7H_2_O_2_ (1 mg); thiamine (1 mg) and riboflavin (1 mg); (II) the medium I plus yeast extract (1 g). Mycelium was harvested on a weighed Whatman No. 41 filter paper, washed with a small quantity of warm distilled water, heated overnight in an oven at about 90 ± 2°C, cooled in a desiccator, and weighed. No further loss in weight was obtained by longer periods of drying.

### 2.2. Enzyme Preparations and Assays

Crude culture filtrate obtained after harvesting mycelium was used for enzyme assays. Culture medium was inoculated in Erlenmeyer flasks (500 mL) by introducing 15 mL of spore suspension into 150 mL of the culture medium. Exoproteinases were isolated from the culture medium after 12 days of growth of the microorganism. Proteins were precipitated with (NH_4_)_2_So_4_ at 80% (w/v) of saturation. The precipitate was separated by centrifugation at 10000 g for 30 min at 4°C. The precipitate was dissolved in water, desalted by gel chromatography on Sephadex G-25, and used for the enzyme assays.

Proteolytic enzyme activity was determined by the Kunitz method [13] using 1% casein, 0.5% azocasein, and 0.5% hemoglobin as substrates. Time of azocasein hydrolysis was 30 min, and for casein and hemoglobin it was 1 h.

 The activity of cysteine proteinases was evaluated in the presence of 25 mM L-cysteine and 1 mM EDTA according to the modified Kunitz method [13]. One unit of proteolytic activity (U) is the amount of enzyme that leads to an increase in optical density in 0.1 at 366 nm (with azocasein) and at 280 nm (with casein and hemoglobin) within 1 min.

Amidase enzyme activity was determined by the method of Erlanger et al. [14] using synthetic and p-nitroanilide substrates: *N*_*α*_-benzoyl-L-arginine *p*-nitroanilide (BAPNA), *N*-carbobenzyloxy-L-alanyl-L-alanyl-L-leucine p-nitroanilide (Z-AALPNA, Bachem, Switzerland), and *N*-succinyl-L-phenylalanine p-nitroanilide (Suc-FPNA), *N*-succinyl-glycyl-glycyl-L-phenylalanine p-nitroanilide (Suc-GGFPNA), L-leucine p-nitroanilide (LPNA), *N*-acetyl-L-alanyl-L-alanyl-L-alanyl p-nitroanilide (Ac-AAAPNA, Bachem, Switzerland). The substrate concentration was 0.5 mM. One unit of amidase activity (AU) was the amount of enzyme that hydrolyzed 1 nmol of the substrate in 1 min.

 In the inhibitor analysis the following inhibitors were used: iodoacetamide (IAA, 1 mM), chloromethylketone tosyl-L-lysine (CMKTL, 1 mM), chloromethylketone tosyl-L-phenylalanine (CMKTP, 1 mM), ethylenediaminetetraacetic acid disodium salt (EDTA, 4.0 mM), DL-dithiothreitol (DTT, 1 mM), phenylmethanesulfonyl fluoride (PMSF, 1 mM), *p*-chloro-mercurybenzoate (PCMB, 1 mM), and diisopropyl fluorophosphate (DIFP, 0.2 mM).

### 2.3. Electrophoresis

Electrophoresis in 20% polyacrylamide gel in the presence of sodium dodecyl sulfate (SDS-PAGE) and ß-mercaptoethanol was performed by the method of Laemmli [15]. Gels were stained with 0.1% solution of Coomassie R-250 in 20% ethanol with 5% formaldehyde.

To obtain the zymograms SDS-PAGE electrophoresis was carried out in the presence of copolymerized substrate (gelatin 0.1%) by the method of Heussen and Dowdle [16]. Protein samples (not more 50 mcg) were applied without prior heating. Upon electrophoresis ending gels were washed with Triton X-100 (2.5%) under vigorous stirring, rinsed with 0.1 M glycine-NaOH buffer pH 7.8, and incubated overnight in the same buffer at room temperature. Gels were then stained with 0.1% amidoschwarz in ethanol : acetic acid : water (3 : 1 : 6) for 1 h and washed with the same solution without the dye. Proteins with proteolytic activity were detected as colorless bands against a deep blue colored background of the stained gelatin.

### 2.4. Protein Content

Protein content was determined by with BSA as a standard using Bradford's modified method [17].

 All experiments and assays were carried out at least in triplicate and the results are presented as mean values obtained with an indication of the standard deviation.

 The chemicals were used of the following companies: azocasein and hemoglobin (Sigma Chemicals Co., USA), casein (Biolar, Latvia), the synthetic substrates mentioned (Sigma, if other not indicated), the synthetic inhibitors mentioned (Sigma), and LMW Calibration Kit (Sigma).

## 3. Results and Discussion

**Figure 1:**
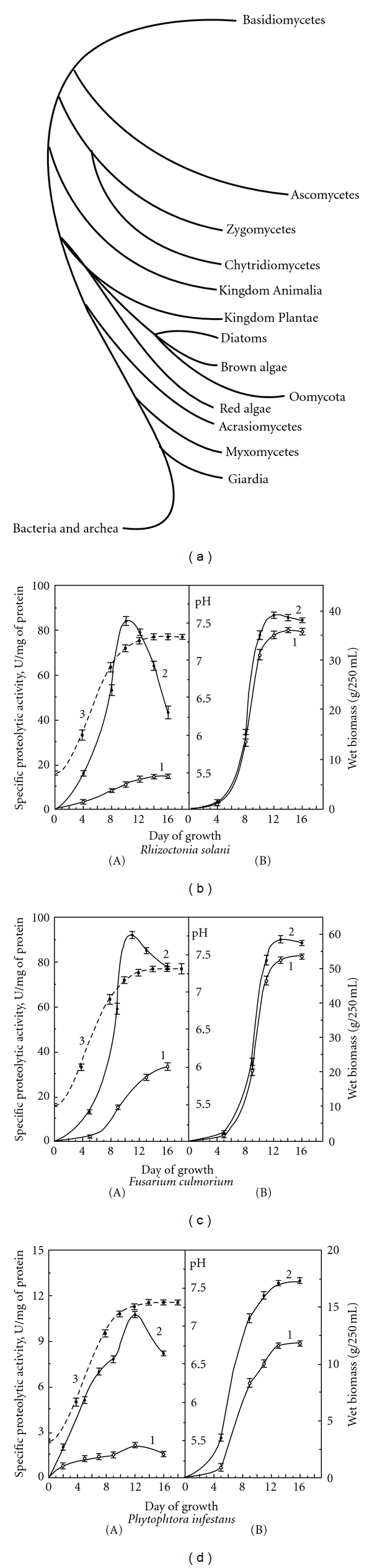
The fungal phylogenetic tree [4] (a) with mapping on it of the exoproteinase activity (A) and wet biomass (B) variations during the growth of *R. solani* (b), *F. culmorum* (c), and *P. infestans* (d) on culture media without (1) and with (2) yeast extract. Curve 3 shows the change in medium pH during the growth of the microorganism.

Culture filtrates of *P. infestans* (Mont.) de Bary, *R. solani*, and *F. culmorum* were tested for the activities of the exoproteinases. The influence of several environmental factors on the production of extracellular proteinases of these microorganisms was studied systematically in controlled batch cultures. Not all of the defined media tested in the study gave production of the examined enzymes, although they all supported fairly good growth (see Figure 1). So we did not observe some changes in yield of proteinases secreted into the culture medium when it was inoculated with these isolates into the semisynthetic culture medium containing KH_2_PO_4_, MgSO_4_, FeSO_4_, thiamin, and riboflavin.

 As the studied pathogen isolates caused the most devastating diseases of potato, we added to the culture media the heat-stable potato tuber proteins. This initiated the secretion of proteinases by fungi *R. solani* and *F. culmorum *(Figures 1(b) and 1(c)). In the case of *P. infestans* the exoproteinase activity remained low and practically unchanged during the growth of the culture, although we observed the biomass increasing (Figure 1(d)). It was shown that the addition of KNO_3_ into the medium lead to a significant decrease in the exoproteolytic activity, indicating the suppression of secretion and possibly synthesis of the exoenzymes. As exoproteinase secretion was inhibited in the presence of nitrate, there was reason to believe that mineral nitrogen regulates adaptation of the pathogens to the environment by a mechanism that, according to the authors of [18], can be attributed to catabolic repression. To study the effect of organic nitrogen on the exoproteinase secretion of the pathogens, yeast extract was extra added into the culture medium. When the yeast extract was added to the culture medium a noticeable increase in the exoproteinase secretion was observed, which was accompanied by accelerated growth of mycelium (Figures 1(b), 1(c), and 1(d)). The yeast extract as an additional source of nutritional substrates for the microorganism apparently acted as an inducer [19]. It is important to point out that the oomycete was able to secret exoproteinases only in the presence of yeast extract. Therefore, we conclude *P*. *infestans* is more exacting in its nutrition for the enzyme production than for growth. It is interesting that there was an observed interdependence for several factors studied. Amongst others, the results showed the clear interaction between nitrogen source and nitrogen concentration. Based on the observed interactions, the selection of environmental factors to increase protease activity is not straightforward, as unexpected antagonistic or synergistic effects can occur. There were some differences in the effect of the environmental parameters on the various proteinase-related phenotypes.

 Not all of the defined media tested in the present study gave production of the examined enzymes, although they all supported fairly good growth. Total proteinase activity increased with the degree of evolutionary “development” of the isolates, which originated from phylogenetically distant fungi belonging to the kingdom true fungi Ascomycetes (*F. culmorum*) and Basidiomycetes (*R. solani*), while *P. infestans* belong to the phylum Oomycota (Figure 1(a)). Traditionally, due to their filamentous growth habit, oomycetes have been classified in the kingdom Fungi. However, modern molecular and biochemical analyses suggest that oomycetes have little taxonomic affinity with filamentous fungi but are more closely related to brown algae (heterokonts) in the stramenopiles, one of several major eukaryotic kingdoms [20–22]. In a series of initial screening experiments of the factors investigated, only medium pH and nitrogen concentration particular strongly affected the extracellular proteinase activities. It was found that the medium pH ranged from slightly acidic to neutral and reached a constant value of 7.2–7.4 after 12 days (Figure 1) during the growth of the isolates. The capacity of pathogenic fungi to support the medium pH did not depend on the composition of the culture media. The dependence of synthesis and secretion of exoproteinases on the medium pH has also been found in some microorganisms [23]. Consequently, the pH can be attributed as one of the factors controlling these processes. Fungi are known to modify the environmental pH to regulate pH [24]. However, fungi normally avoid natural habitats with unsuitable pH, possibly because of the metabolic costs of this type of adjustments in competition with more specifically pH-adapted microorganisms. Finally, the proteolytic enzyme activity is known to be strongly pH dependent, so in order to have effective protein degradation the pH optimum of the proteolytic enzymes should ideally match the pH of their habitat. We observed that the pH values of the medium affected the growth of the isolates (Figure 1, curves 3).

 It was shown that the secretion of proteinases depended on the cultivation temperature. When the culture was grown at 28°C a decrease in the proteolytic amidase activity was observed. The temperature of 21°C was optimal for the production of exoproteinases by the isolates, and apparently corresponded to the temperature regime of its habitat in the natural environment. This temperature optimum for the studied pathogen and its inability to grow at higher temperatures may reflect its distribution in natural habitats that are buffered against higher temperatures. The study of Allain-Boulé et al. [25] reported temperature optimum of 20–25°C for several strains of *Pythium attrantheridium* isolated from cavity spot lesions on carrots and on apple and cherry seedlings. Similarly, *in vitro* growth of *Pythium splendens*, a species that causes a root disease of carambola in southern Florida, decreases at temperatures above 30°C  [26].

 The exoenzymes secreted by fungi were the most effective at neutral and slightly alkaline pH values. So, exoproteinases of *F. culmorum* were characterized by the maximum of proteolytic activity at pH 8.0, and of *R. solani* at pH 8.5. The highest level of exoproteinase activity of *P. infestans* was observed at neutral pH values and was characterized by a maximum at pH 7.0. The second some increase of the enzyme activity of *P. infestans* was exhibited at slightly alkaline pH indicating the presence of proteinases with pH optimum of action in the region from 8 to 9.

**Figure 2:**
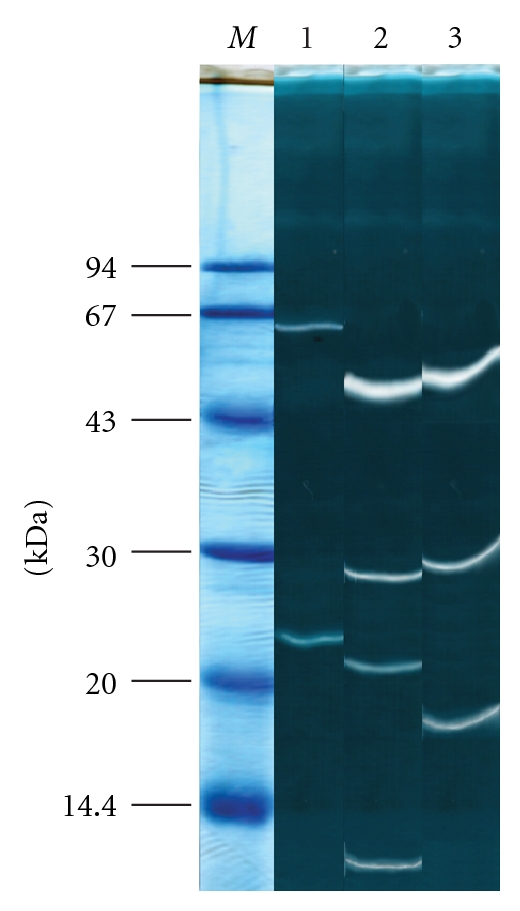
Gelatinous zymograms of exoproteinases obtained by SDS-PAGE of partially purified culture media after 12 days of growth of *R. solani* (lane 1), *P. infestans *(lane 2), and *F. culmorum* (lane 3). Lane *M* represents the molecular mass (kDa) markers as follows: phosphorylase b (94), bovine serum albumin (67), egg albumin (43), carboanhydrase (30), Kunitz soybean trypsin inhibitor (20,1), and lactalbumin (14.4). About 50 mcg of the protein were added onto the lines 1–3.

 It was indicated by SDS-PAGE that all studied isolates secreted three or more proteins with proteolytic activity (Figure 2). They had molecular masses ranging from 12 to 65 kDa. *Fusarium culmorum* and *P. infestans* predominantly produced 29- and 49-kDa proteinases, and *R. solani* secreted a 67- and 22-kDa proteinases (Figure 2). Exoproteinases of all three pathogens showed low activity toward casein and hemoglobin, while activity in azocasein assay was much higher (Table 1).

Table 2 presents data of the exoenzyme activity dependence on the substrate used. There are clear catalytic differences between subtilisins and trypsins, in their substrate specificities can allow their distinction. It is evident that P. infestans exoproteinases most effectively hydrolyzed BAPNA (a substrate for trypsin-like proteinases) and to a lesser extent Z-AALPNA (a substrate for subtilisin-like proteinases). At the same time, the exoproteinases did not act on the substrates for chymotrypsin- and elastase-like proteinases (Suc-GGFPNA and Ac-AAAPNA, resp.), as well as for aminopeptidases (LPNA). The enzymes secreted by *F. culmorum* hydrolyzed Z-AALPNA very efficiently and to a lesser extent BAPNA. They showed low activity toward substrates for chymotrypsin- and elastase-like proteinases, and for aminopeptidases as well. For the exoenzymes secreted by *R. solani* the profile depended on the culture medium composition (Table 3). BAPNA was hydrolyzed most efficiently if the yeast extract was absent, but Z-AALPNA was significantly less efficiently hydrolyzed. Specific substrates for chymotrypsin- and elastase-like proteinases and for aminopeptidases were hydrolyzed poorly if at all. The addition of yeast extract led to a change in the proteinase spectrum: Z-AALPNA was hydrolyzed most effectively, but BAPNA was much more poorly hydrolyzed (more than five times, see Table 3). Analysis of the data on the effect of synthetic substrates specific to certain groups of proteinases to the exoenzyme activity of the oomycete and the fungi indicated that *P. infestans* secreted predominantly serine and metalloproteinases, and the enzymes of serine type are trypsin- and subtilisin-like proteinases. In the case of *F. culmorum* the exoproteinases are represented mainly by subtilisin- and trypsin-like enzymes. The exoproteinase profile of *P. infestans* and *F. culmorum* were not dependent on medium composition.

 Exoenzymes of the fungus *R. solani* were represented by serine-type proteinases too. When *R. solani* was grown without yeast extract, trypsin-like serine proteinases were secreted mainly, including SH-dependent serine enzymes. The subtilisin-like proteinase activity was significantly lower (Table 3). In the presence of yeast extract the composition of *R. solani* exoenzymes was enriched in subtilisin-like proteinases whereas the content of trypsin-like enzymes was decreased significantly (Table 3). The presence of a reducing agent (L-cysteine with EDTA) did not affect the proteolytic activity of the fungal exoproteinases in the azocasein assay, which indicated the absence of cysteine exoproteinases in the growth medium.

 The interaction of different synthetic inhibitors with exoproteinases secreted by the three microorganisms was studied (Table 4). EDTA, which has often been used as an indicator of metalloproteinases, had some effect only upon the total proteolytic activity of the exoenzymes of *P. infestans.* The proteolytic activity of *P. infestans* exoproteinases was reduced by almost twofold in the presence of L-cysteine with EDTA. This indicated the presence of exometalloproteases whose activity was inhibited by EDTA (Table 4). This was confirmed by increase in the *P. infestans* exoproteinase activity by 50% in the presence of 1 mM CaCl_2_ at pH 7.0. It is well known that metalloproteinases of microorganisms are activated in the presence of calcium ions [27]. PMSF inhibited effectively; this suggests that all the exoenzymes are serine proteinases. Analysis of chloromethylketone treatment showed that the serine proteinases were trypsin-like (Table 4). So the data obtained in the experiments with synthetic inhibitors confirmed that the oomycete *P. infestans* secreted serine- and metalloproteinases predominantly and that serine-type enzymes were trypsin- and subtilisin-like proteinases. The* F. culmorum* exoproteinases were mainly subtilisin- and trypsin-like enzymes. Treatment with mercuric chloride significantly reduced the amidase proteolytic activities of the exoenzyme (Table 4). That serves as once more confirmation of the presence of serine proteinases in the culture medium of *F. culmorum*. The results of the azocasein assays (Table 4) show that inhibitions of aspartate and cysteine proteinases were small or absent.

**Figure 3:**
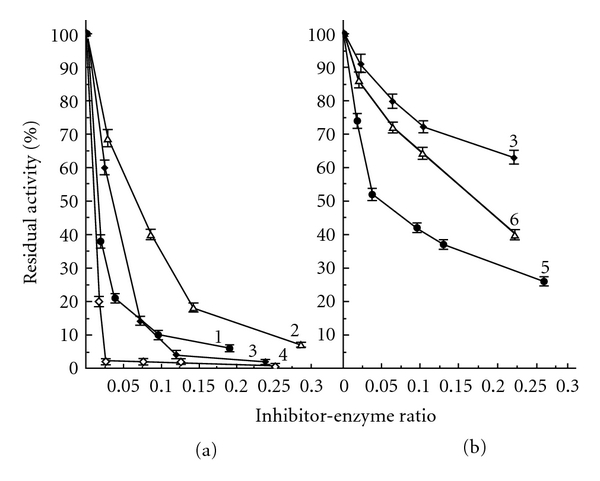
Effect of protein proteinase inhibitors on amidase activities (%) of *R. solani* (a) and *F. culmorum* (b) exoproteinases. 1—trypsin inhibitor from potato tubers [27], 2—chymotrypsin I inhibitor [30], 3—SKTI, 4—trypsin inhibitor from honey locust seeds [31], 5—subtilisin inhibitor from potato tubers [32], 6—SBBI. For the amidase activity measurements BAPNA and Z-AALPNA were used as the substrates, respectively.

 The interaction of exoproteinases secreted by *R. solani* and *F. culmorum* with the natural protein serine proteinase inhibitors isolated from potato tubers and legume seeds was also studied (Figure 3). The activity of *R. solani* exoproteinases was inhibited most effectively by specific trypsin inhibitors from potato tubers and from honey locust seeds as well as soybean Kunitz trypsin inhibitor (SKTI) (Figure 3(a), curve 1, 3, and 4). The interaction of potato chymotrypsin inhibitor I with exoproteinases was much weaker (Figure 3(a), curve 2). The mentioned assumption about the trypsin-like activity of *R. solani* exoproteinases grown without yeast extract was confirmed. SKTI and soybean Bowman-Birk inhibitor (SBBI) acted on exoproteinases of *F. culmorum* much more weakly (Figure 3(b), curve 3 and 6). However, the specific subtilisin inhibitor from potato tubers inhibited effectively their activity, reducing it by more than 60% (Figure 3(b), curve 5). This indicates that the subtilisin-like enzymes constitute a significant part of the *F. culmorum* exoproteinases. Similar results were obtained in the study of protein inhibitor action on the enzymes secreted by *P. infestans* (data not presented). The data obtained in the experiments confirmed belonging of exoproteinases of the fungi and oomycete to the chymotrypsin clan of proteolytic enzymes [6]. It should be noted that potato subtilisin inhibitor suppressed *in vitro* growth and development of *F. culmorum *macroconidia and *P. infestans* zoospores [27]. We can assume that the secreted exoproteinases of the phytopathogenic microorganisms are a factor of their pathogenicity.

 Thus, the inhibitor analysis of the major extracellular proteinases of pathogenic fungi *R. solani* and *F. culmorum* and the oomycete *P. infestans* showed that they belong to the group of serine proteinases mainly. The substrate specificity of the proteinases and their sensitivity to synthetic and natural inhibitors suggested that the enzymes of *F. culmorum* and *P. infestans* are trypsin-like and subtilisin-like proteinases. The exoenzymes of *R. solani* depended on the culture medium composition, especially on the form of nitrogen nutrition. When *R. solani* grows as a saprobe the exoenzyme was represented by subtilisin-like proteinases. Although *R. solani* has been isolated only from potato tissues, it can be thought to persist as a saprobe that lives on plant debris, as evidenced by the increase in subtilisin-like activity. The secretion of the trypsin-like proteinases in culture can be due to their participation in tissue degradation or aid to infection by destroying pathogenesis-related proteins or other nonstructural molecules. It is intriguing to speculate that exoproteolytic competence of the ascomycete *R. solani* allowed growth on a greater variety of living and nonliving proteinaceous substrates. In reviewing the protease data it is important to recognize that only those enzymes that retain activity after sample electrophoresis are displayed by SDS-substrate-PAGE, whereas the azocasein assays report the sum of the activities of all of the proteases present in the sample. This explains the differences under the condition of the electrophoretic separation (Figure 2) and the effects of inhibitors on total protease activity shown in Table 4. This may have been a component allowing niche differentiation between the ascomycetes and the basidiomycetes, which will have adapted the former to pathogenicity to animals or may have derived from adaptation to pathogenicity. In any event, the fact that two families of subtilisins radiated in the early ascomycetes suggest that these fungi had a lifestyle that selected for multiple proteinase activities.

## 4. Conclusion

The range of nutritional sources utilized by a certain fungus is regarded as a consequence of diverse molecular, cellular, and ecological factors. Many of the enzymes secreted by pathogenic fungi can affect their relationships with plant and animal hosts. This suggests that differences in the properties of the enzymes provide selective advantages in different habitats. The serine proteinases are very widespread in nature and are involved in a wide variety of biological processes. Enzymes belonging to this class vary significantly in substrate specificity, which can correspond to the requirements of fungal ecological niches [7]. According to the opinion of Hu and St. Leger R. J. [28], the fragmentary distribution of trypsins among fungi indicates that their phylogenetic distribution may be greater in the early fungi than in modern ones.

 Our data suggest that different nutritional sources can be important for the differential production of serine proteinases. The multiple subtilisins in pathogenic fungi could play different roles in pathogenesis, increase adaptability and host range, or have different functions in survival in various ecological habitats outside the host. Like subtilisins, the trypsins are inducible by environmental cues [9, 29]. Thus, there are several mechanisms available for different strains to adapt enzyme activities to their specific needs on their particular hosts. The differential production of these classes of proteolytic enzymes suggest that substrate specificity may be important and that tradeoffs may prevent the simultaneous upregulation of both classes of enzymes. It appears total proteinase activity increases with the degree of evolutionary “advancement” of the fungus. These exceptional phylogenies could reflect convergent evolution through which phylogenetically distinct enzymes evolved to share significant similarity, perhaps by targeting similar substrates.

 Despite few morphological similarities, phylogenetic analysis have shown that there are more similarities in the exoproteinase composition between the oomycete *P. infestans* and the ascomycete *F. culmorum *although they are more distant relatively than the distance between the ascomycete and basidiomycete *R. solani*. Our study also suggests that the *in vitro* behavior of these species cannot be directly related to the ecological niche from which they have been isolated. This difference between the proteinases can reflect the physiological difference between their nutritional environments (saprotroph and phytopathogen) [9].

 Fungi normally produce a wide range of proteolytic enzymes to degrade protein substrates. However, differences in the properties of the proteinases found in the studied organisms were unlikely to be caused by variations in food substrate composition only as all our experiments involved the same culture media. It seems likely, therefore, that the proteinase compositions that we observed have a significant genetic component.

## Figures and Tables

**Table 1:** Proteinase activities secreted by the three species studied in azocasein assay.

Species	Mycelia wet weight, g/250 mL of medium	Specific proteinase activity, U/mg of protein
*P. infestans*	17.5 ± 0.04	4.5 ± 0.14
*F. culmorum*	58.3 ± 0.18	27.2 ± 1.07
*R. solani*	38.5 ± 0.05	22.4 ± 0.92

**Table 2:** Exoproteinase activity (U, nmol/mg of substrate per minute) of the oomycete *P. infestans,* fungi *R. solani,* and *F. culmorum* in hydrolysis of *p-*nitroanilides of amino acids and tripeptides (substrate concentration was 0.5 mM).

Substrate	Exoproteinases of *P. infestans *	Exoproteinases of *R. solani *	Exoproteinases of* F. culmorum *
BAPNA	4.2 ± 0.17	2.7 ± 0.07	1.9 ± 0.06
Z-AALPNA	2.1 ± 0.08	0.5 ± 0.02	6.7 ± 0.23
Suc-FPNA	0 ± 0.00	0 ± 0.00	0.4 ± 0.01
Suc-GGFPNA	0 ± 0.00	0 ± 0.00	0.5 ± 0.01
LPNA	0 ± 0.00	0.1 ± 0.01	1.1 ± 0.03
Ac-AAAPNA	0 ± 0.00	0.1 ± 0.01	1.0 ± 0.02

**Table 3:** Total exoproteinase activity of *R. solani* grown on medium without and with yeast extract.

Substrate	Specific activity, U/mg of protein/min	
	with 1% yeast extract	without yeast extract
Azocasein	83.3 ± 2.34	15.0 ± 0.56
BAPNA	26.0 ± 0.88	10.0 ± 0.31
Z-AALPNA	40.0 ± 1.38	1.8 ± 0.06

**Table 4:** Effect of synthetic inhibitors on the activity of exoproteinases secreted by *P. infestans*, *F. culmorum,* and *R. solani* (concentration of DIFP was 0.2 mM, EDTA was 4.0 mM, and the other inhibitors were 1.0 mM). Activity without inhibitors was taken as 100%.

Inhibitor	Substrate								
	*P. infestans*, % of residual activity			*R. solani*, % of residual activity			*F. culmorum,* % of residual activity		
	Azocasein	BAPNA	Z-AALPNA	Azocasein	BAPNA	Z-AALPNA	Azocasein	BAPNA	Z-AALPNA
DIFP	na*	42.0 ± 1.4	na	na	na	na	na	na	na
PMSF	100 ± 0.1	100 ± 0.2	0 ± 0.0	61.0 ± 2.4	96.0 ± 3.1	0 ± 0.0	35.0 ± 0.8	17.4 ± 0.6	78.0 ± 2.1
CMKTL	na	4.0 ± 0.2	95.0 ± 3.5	95.0 ± 3.8	70.0 ± 1.9	72.0 ± 2.5	84.0 ± 3.1	50.0 ± 1.8	52.0 ± 2.4
CMKTP	na	52.0 ± 2.1	91.0 ± 2.9	82.0 ± 3.1	84.0 ± 2.8	85.0 ± 4.1	80.0 ± 3.8	46.0 ± 2.0	100 ± 0.1
EDTA	20.3 ± 0.5	41.0 ± 1.6	0 ± 0.0	89.0 ± 3.6	93.0 ± 3.5	90.0 ± 3.6	112.0 ± 3.9	99.0 ± 0.6	99.0 ± 0.4
PCMB	95.1 ± 3.2	69.0 ± 2.7	100 ± 0.1	76.0 ± 2.7	95.0 ± 3.2	80.0 ± 2.6	61.0 ± 1.7	100 ± 0.1	100 ± 0.2
IAA	100 ± 0.1	90.1 ± 3.8	100 ± 0.2	100 ± 0.1	93.0 ± 2.9	100 ± 0.1	97.0 ± 2.1	101.6 ± 0.8	101.2 ± 0.9
DTT	100 ± 0.1	93.4 ± 4.2	84.2 ± 2.8	66.0 ± 2.3	129.1 ± 4.4	91.0 ± 4.0	82.0 ± 2.5	98.4 ± 1.0	99.2 ± 0.5
Mercuric chloride	65.4 ± 2.4	57.2 ± 2.2	27.4 ± 1.3	79.0 ± 3.0	95.0 ± 2.5	89.0 ± 2.9	53.0 ± 2.0	34.0 ± 1.3	55.2 ± 2.2

*na—not assayed.
